# *IL10* promoter variants are associated with gene expression but they are not markers of susceptibility to acute coronary syndrome

**DOI:** 10.1038/s41598-024-64097-2

**Published:** 2024-06-08

**Authors:** Texali Candelaria Garcia-Garduño, Jorge Ramón Padilla-Gutiérrez, Maricela Aceves-Ramírez, Brenda Parra-Reyna, Héctor Enrique Flores-Salinas, Emmanuel Valdes-Alvarado, Denisse Stephania Becerra-Loaiza, Antonio Quintero-Ramos, Iliannis-Yisel Roa-Bruzón, Andrea de la Cruz, Yeminia Valle

**Affiliations:** 1 Instituto de Investigación en Ciencias Biomédicas (IICB), Centro Universitario de Ciencias de la Salud (CUCS), Universidad de Guadalajara (UDG), Guadalajara, Jalisco México; 2 Doctorado en Genética Humana (DGH), Centro Universitario de Ciencias de la Salud (CUCS), Universidad de Guadalajara (UDG), Guadalajara, Jalisco México; 3 Especialidad en Cardiología, Unidad Médica de Alta Especialidad, Centro Médico Nacional de Occidente (CMNO), Departamento de Cardiología, Instituto Mexicano Del Seguro Social (IMSS), Guadalajara, Jalisco Mexico; 4 Laboratorio de Inmunología, Departamento de Fisiología, Centro Universitario de Ciencias de la Salud, Universidad de Guadalajara, Guadalajara, México; 5 Unidad de Investigación Biomédica 02, Hospital de Especialidades, Centro Médico Nacional de Occidente, Instituto Mexicano del Seguro Social, Guadalajara, México; 6 Laboratorio de Bromatología, Departamento de Ciencias Biomédicas, Centro Universitario de Tonalá, Universidad de Guadalajara (UDG), Guadalajara, Jalisco México; 7 Departamento de Aparatos y Sistemas II, Universidad Autónoma de Guadalajara, Zapopan, Jalisco, México

**Keywords:** Gene expression, Haplotypes, Immunogenetics, Population genetics

## Abstract

Interleukin-10 (IL-10) is an immunomodulatory cytokine that plays a pivotal role in the pathogenesis of acute coronary syndromes (ACS). Here, we evaluated the role of *IL10* promoter variants as markers for ACS susceptibility in Western Mexican patients as well as its association with *IL10* mRNA and IL-10 plasma levels. Three promoter variants (− 1082 A > G, − 819 T > C and − 592 A > C) were analyzed in 300 ACS patients and 300 control group (CG) individuals. *IL10* relative gene expression was evaluated in peripheral blood mononuclear cells (PBMC) and IL-10 levels were quantified in plasma. The allelic, genotypic and haplotypic frequencies did not show significant differences between groups. ACS patients had sevenfold higher mRNA *IL10* level compared to CG (p = 0.0013). Homozygous C/C carriers in both − 819 T > C and − 592 A > C variants had 0.4-fold higher *IL10* mRNA expression than heterozygous and polymorphic allele homozygous genotypes (p = 0.0357) in ACS group. There were significant differences in plasma IL-10 levels in CG and ACS group (1.001 vs 1.777 pg/mL, p = 0.0051). The variants were not markers of susceptibility to ACS in Western Mexican individuals. ACS patients showed higher *IL10* expression than CG individuals which could be mediated by − 819 T > C and − 592 A > C variants and pharmacotherapy.

## Introduction

Acute coronary syndrome (ACS) is the leading cause of cardiovascular disease morbimortality worldwide^1^. ACS comprises a spectrum of conditions including unstable angina (UA) and myocardial infarction in its STEMI (ST-segment elevation myocardial infarction) and NSTEMI (Non-ST segment elevation myocardial infarction) electrocardiographic presentations. ACS is the clinical manifestation of atherosclerotic plaque rupture where inflammation plays a central role. In atherosclerotic lesions, several alterations can be observed directed by an exacerbation of the proinflammatory response that propitiates progression to ACS. There is evidence that interleukin-10 (IL-10) exerts diverse antiatherogenic effects throughout different stages of atherosclerosis and in the thrombus formation^2^. Signaling mediated by IL-10 and its receptor (IL-10R) causes a decrease in the expression profile of proinflammatory genes^3^. Additionally, under IL-10 stimulus a polarization of macrophages to the M2 (anti-inflammatory) subtype occurs^4^. Also, IL-10 can inhibit the expression of Monocyte chemoattractant protein-1 (MCP-1), Intercellular Adhesion Molecule-1 (ICAM-1)^5^ and chemokines^6^ preventing the recruitment and differentiation of monocytes into macrophages^7^. George et al. reported that patients with vulnerable plaques have decreased IL-10 levels compared to those with stable plaques^8^, thus, the relevance of a correct expression and function of this cytokine is essential in the progression of culprit lesions. IL-10 is the major immunomodulatory cytokine. The special physiological relevance of this cytokine relies in the prevention and limitation of specific and nonspecific immune reactions and consequently tissue damage^9^. The *IL10* gene is located on the long arm of chromosome 1 (1q32.1). It consists of 5.1 kpb, contains five exons and four introns^10^. The promoter region of the *IL10* gene is characterized as a highly polymorphic region. Biallelic variants at positions -1082 A > G (rs1800896), − 819 T > C (rs1800871) and − 592 A > C (rs1800872) are located in the proximal promoter region. These variants have been associated with alterations in transcriptional activity and plasma levels of the cytokine in diverse pathologies of inflammatory nature, including ACS^11–14^. Considering the central role of the IL-10 in the pathogenesis of ACS, the aim of the study was (1) to evaluate the role of *IL10* promoter variants as markers for ACS susceptibility in Western Mexican patients, (2) to analyze the association of promoter SNVs with *IL10* mRNA levels and (3) to evaluate IL-10 plasma levels in ACS patients.

## Results

Demographic and clinical characteristics of ACS patients and CG individuals are described in Table 1. The median age of the ACS group was 64 years and, in the control group it was 57 years. The male gender was the most affected (ratio 3:1) in the ACS group. Of the clinical spectrum, 61% were STEMI, 22% NSTEMI, and 17% were UA. The most prevalent risk factor in both groups was High Blood Pressure (HBP).

**Table 1:** Demographic and clinical characteristics of the ACS patients and CG individuals.

Demographic characteristic	CG, *n* (%)	ACS group, *n* (%)	p
Cardiovascular risk factor			
Age (median, IQR)	57 (51–63)	64 (57–70)	< 0.0001
Male	143 (48)	215 (72)	< 0.0001
Female	157 (52)	85 (28)	< 0.0001
High blood pressure	100 (33)	214 (71)	< 0.0001
Dyslipidemia	65 (22)	148 (49)	< 0.0001
T2DM	65 (22)	172 (57)	< 0.0001
Smoking	66 (22)	143 (48)	< 0.0001
Overweight	85 (28)	116 (39)	0.0043
Obesity	81 (18)	80 (27)	0.0084
Sedentary lifestyle	88 (29)	181 (60)	< 0.0001
Reinfarction	–	53 (18)	–
Medication			
ASA	3 (1)	287 (96)	< 0.0001
Clopidogrel	–	(92)	–
Metformin	18 (6)	21 (7)	0.6196
Insulin	–	12 (4)	–
Antihypertensive	11 (4)	61 (20)	< 0.0001
Statin	21 (3)	285 (95)	< 0.0001
Bezafibrate	5 (2)	8 (3)	0.6043
Losartan	21 (7)	54 (18)	< 0.0001
β-blocker	6 (2)	180 (60)	< 0.0001
Enalapril	6 (2)	135 (45)	< 0.0001
Captopril	–	51 (17)	–
Isosorbide	–	72 (24)	–
Amlodipine	46 (12)	30 (10)	0.4341
Heparin	–	42 (14)	–
Enoxaparin	–	201 (67)	–

Obesity and overweight were defined according to the World Health Organization criteria^15^.

*CG* control group, *ACS* acute coronary syndrome, *IQR* interquartilic range, *T2DM* type 2 diabetes mellitus, ASA acetyl salycilc acid, and *ACEI* angiotensing converting enzyme inhibitors.

### Genetic analysis

According to HWE analysis, both groups were at equilibrium at each locus (p > 0.05). The polymorphic allele for each variant was the least prevalent in both groups. For − 819 T > C and − 592 A > C loci, the heterozygous genotype was the most common. In the case of − 1082 A > G variant, the T/T genotype was the most frequent one. The allelic and genotypic frequencies of the variants analyzed did not show significant differences between groups (Table 2). Also, none of the genetic models showed association with the susceptibility to developing ACS in Western Mexican individuals.

**Table 2:** Distribution of* IL10* gene promoter variants among ACS patients and CG individuals.

SNV	Genotype	ACS (n = 300), n (%)	CG (n = 300), n (%)	OR (95% CI)	p
− 1082 A > G (rs1800896)					
Allele	A	426 (71)	420 (70)	1.0	–
	G	174 (29)	180 (30)	0.94 (0.73–1.22)	0.68
pHWE		0.57	0.41		
Codominant	A/A^¥^	153 (51)	150 (50)	1.0	–
	A/G	120 (40)	120 (40)	0.98 (0.70–1.38)	0.90
	G/G	27 (9)	30 (10)	0.43 (0.50–1.55)	0.66
Dominant	A/A	153 (51)	150 (50)	1.00	
	A/G + G/G	147 (49)	150 (50)	0.96 (0.69–1.33)	0.8
Recessive	A/A + A/G	273 (91)	270 (90)	1.00	
	G/G	27 (9)	30 (10)	0.88 (0.51–1.53)	0.65
− 819 T > C (rs1800871)					
Allele	C	369 (62)	369 (62)	1.0	–
	T	231 (38)	231 (38)	1.02 (0.80–1.29)	0.84
pHWE		0.54	0.33		
Codominant	C/C^¥^	111 (37)	117 (39)	1.0	–
	C/T	147 (49)	135 (45)	1.21 (0.85–1.72)	0.47
	T/T	42 (14)	48 (16)	0.324 (0.56–1.50)	0.74
Dominant	C/C	111 (37)	117 (39)	1.0	–
	C/T + T/T	189 (63)	183 (61)	1.14 (0.82–1.59)	0.44
Recessive	C/C + C/T	258 (86)	252 (84)	1.0	–
	T/T	42 (14)	48 (16)	0.86 (0.55–1.35)	0.51
− 592 A > C (rs1800872)					
Allele	C	363 (60)	369 (61)	1.0	–
	A	237 (40)	231 (39)	0.96 (0.76–1.21)	0.75
pHWE		0.62	0.39		
Codominant	C/C^¥^	108 (36)	117 (39)	1.0	–
	C/A	147 (49)	135 (45)	1.19 (0.84–1.70)	0.57
	A/A	45 (15)	48 (16)	0.06 (0.62–1.64)	0.94
Dominant	C/C	108 (36)	117 (39)	1.0	–
	C/A + A/A	192 (64)	183 (61)	1.14 (0.82–1.60)	0.44
Recessive	C/C + C/A	255 (85)	252 (84)	1.0	–
	A/A	45 (15)	48 (16)	0.91 (0.58–1.42)	0.67

*SNV* single nucleotide variant, *pHWE* hardy–weinberg equilibrium p-value, *CG* control group, *ACS* case group, OR odds ratio, *CI* confidence interval.

^¥^Reference category.

Because these SNVs have been described to be closely related, LD analysis was performed. The variants showed a high linkage disequilibrium (Dʹ = 0.989, r^2^ = 0.915, p < 0.0001) and three major haplotypes (ATA, GCC, and ACC) were observed. The order of the SNVs in the haplotypes is indicated according to their position in the chromosome (− 1082 A > G, − 819 T > C and − 592 A > C). The haplotype frequencies of the studied groups are shown in Table 3.

**Table 3:** Haplotype analysis of the variants in the *IL10* gene promoter among ACS patients and CG individuals.

Haplotype	ACS (%), n = 300	CG (%), n = 300	OR (95% IC)	p
ATA^¥^	114 (38)	111 (37)	1.0	–
ACC	93 (31)	93 (31)	1.005 [0.784–1.289]	0.9691
GCC	84 (28)	87 (29)	0.958 [0.743–1.234]	0.7377

Haplotypes with frequency < 3% (GTA, ACA, ATC, GCA) were not considered in the analysis. The GTC haplotype was not observed in any of the study groups.

*CG* control group, *ACS* case group, *OR* odds ratio, *CI* confidence interval.

^¥^Reference Category.

### *IL10* relative expression analysis

**Figure 1:**
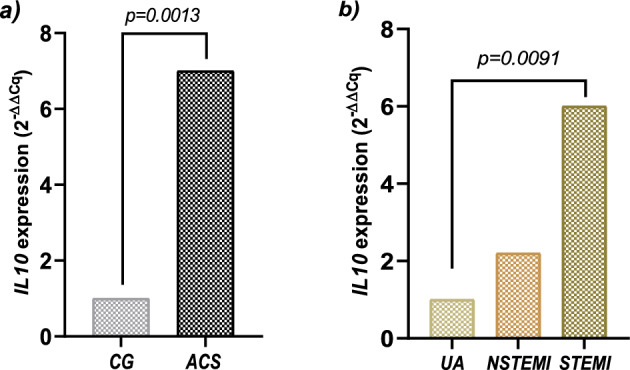
Gene expression levels in the CG and the ACS group. (**a**) Comparison of *IL10* mRNA levels observed in the CG and the ACS group. P-value was determined by the Mann–Whitney *U* test. (**b**) Comparison of *IL10* gene expression levels by clinical spectrum in the ACS group. Patients were stratified according to clinical presentation. Data was analyzed by the comparative 2^−ΔΔCq^ method and are presented as fold-change. Data was analyzed by Kruskal–Wallis test and adjusted p-value obtained from Dunn’s post-hoc test.

The relative expression of *IL10* mRNA was evaluated in PBMC from patients with ACS (n = 28) and CG (n = 28). The CG and the ACS group were matched by age for this analysis. The results were analyzed using the 2^−ΔΔCq^ method. The relative expression of *IL10* in ACS patients was sevenfold higher than in the CG (p = 0.0013, Fig. 1a). Subsequently, patients were stratified by clinical presentation (9 UA, 9 STEMI and 10 NSTEMI patients). According to the 2^−ΔΔCq^ analysis, it was observed that patients with STEMI presented sixfold higher expression of *IL10* compared to patients with UA (p_adjusted_ = 0.0091, Fig. 1b). No significant differences in *IL10* gene expression were observed when comparing UA *vs* NSTEMI (p_adjusted_ = 0.2244) neither comparing NSTEMI *vs* STEMI patients (p_adjusted_ = 0.0544).

**Figure 2:**
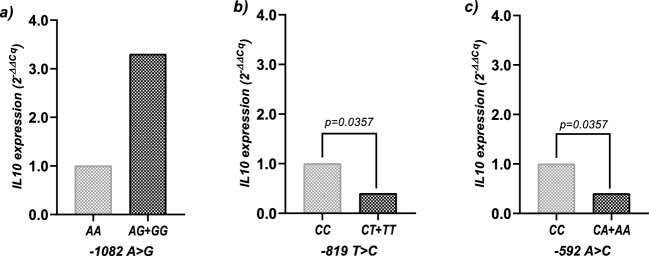
Comparison of *IL10* gene expression levels in the ACS group under the dominant model of inheritance. (**a**) Comparison of *IL10* mRNA levels for − 1082 A > G variant. A/A = 15 and A/G + G/G = 13 individuals. (**b**) Comparison of *IL10* mRNA for − 819 T > C variant. T/T = 9 and C/T + T/T = 19. (**c**) Comparison of *IL10* mRNA levels for − 592 A > C variant. C/C = 8 and C/A + A/A = 20. Data was analyzed by the comparative 2^−ΔΔCq^ method and are presented as fold-change. P-value was determined by the Mann–Whitney *U* test.

To evaluate the influence of these three *IL10* promoter variants on its corresponding mRNA level, we analyzed the *IL10* gene expression under the dominant model of inheritance. In the ACS group we found that carriers of the C/C genotype in loci − 819 T > C and − 592 A > C had higher expression in comparison to the heterozygous and polymorphic allele homozygous genotypes (0.4-fold times, p = 0.0357, Fig. 2b,c). In the case of − 1082 A > G variants, in this same context, we did find a trend towards higher levels in carriers of the A/G and G/G genotypes comparing with A/A carriers, however, no statistical differences were found (p = 0.0762, Fig. 2a). It is worth noting that, since these three variants show an important LD, a more appropriate analysis should be the analysis of haplotypes regarding gene expression. However, due to the relative low frequency of the polymorphic allele of each variant, we performed this analysis under the dominant model of inheritance.

### Plasma concentration of IL-10

**Figure 3:**
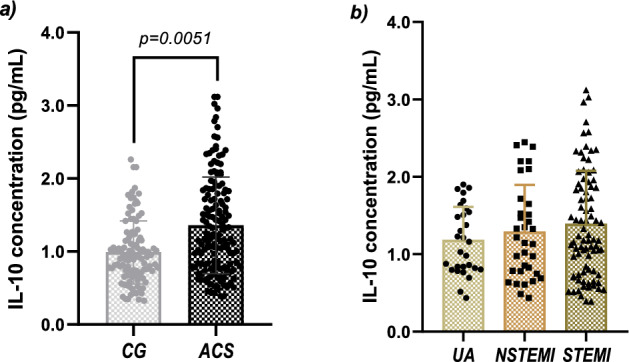
Comparison of IL-10 plasma levels. (**a**) Comparison of IL-10 plasma levels between the CG and the ACS group. Data is presented as median (IQR). CG: 1.011 (0.741–1.546 pg/mL and ACS: 1.325 (0.823–1.918) pg/mL. P-value was determined by the Mann–Whitney *U* test. (**b**) Comparison of IL-10 plasma levels among the clinical entities of ACS. UA = 1.221 (0.833–1.650) pg/mL, NSTEMI = 1.181 (0.781–1.617) pg/mL, and STEMI = 1.209 (0.776–1.878) pg/mL. Data was analyzed by Kruskal–Wallis test and adjusted p-value was obtained from Dunn’s post-hoc test.

Quantification of plasma IL-10 levels was performed in 126 CG individuals (61 female and 65 male samples) and 157 ACS patients (57 female and 100 male). Also, we measured the baseline levels of IL-10 plasma levels in a pool of 78 healthy western Mexican individuals that conformed a reference group (RG), as described in detail in "Material and methods" section. We observed significant differences in plasma concentration of IL-10 in the CG and the ACS group (1.001 vs 1.777 pg/mL, p = 0.0051, Fig. 3a). Considering the reference range (1.0441–1.8704 pg/mL), the ACS group had a median concentration similar to the RG (1.777 vs 1.5577 pg/mL, respectively, p = 0.1435). Nevertheless, the CG had significant lower levels (1.001 vs 1.557 pg/mL, p = 0.0002) when comparing to the RG. In the ACS group, 85 STEMI, 35 NSTEMI, and 35 UA samples were analyzed. No statistical significance was found when analyzing the IL-10 cytokine concentration according to the clinical spectrum (1.209, 1.181 and 1.221 pg/ml, respectively, p = 0.8302, Fig. 3b), contrary to what was observed in regard to the expression of *IL10* mRNA, where the STEMI entity showed the greater expression (Fig. 1b).

At the time of blood sampling, the individuals were under pharmacological treatment. As the drugs that were part of the medical treatment could influence *IL10* mRNA and IL-10 levels, we performed a linear regression analysis in both groups. For gene expression analysis, in the ACS group we observed that some drugs influenced mRNA and plasma IL-10 levels, as shown in Table 4. In the CG, any drug (p > 0.05) showed an effect over *IL10* mRNA or IL-10 levels according to the linear regression model.

**Table 4:** Drugs of the pharmacological treatment in ACS patients.

mRNA *IL10*			Plasma IL-10 levels		
Drug	β	p	Drug	β	p
Bezafibrate	− 2.35	0.0207	Bezafibrate	6.74	< 0.0001
Amlodipine	1.88	0.0424	ASA	− 9.45	< 0.0001
Isosorbide	2.16	0.0061	Clopidogrel	4.12	0.0040
Metformin	− 1.60	0.0456	Furosemide	2.32	0.0527
β-blocker	− 1.52	0.0603			
Enoxaparin	− 1.95	0.0957			

mRNA model adjusted R^2^ = 0.3945, p = 0.0015, plasma IL-10 adjusted R^2^ = 0.2609, p < 0.0001.

*β* slope of the regression line, *ASA* acetyl salicylic acid.

Regarding cardiovascular risk factors (CRF) and IL-10 plasma levels, body mass index (BMI) category (β = 0.59, p = 0.0129) and metabolic syndrome (β = − 1.58, p = 0.0464) influenced IL-10 plasma levels in the CG (adjusted R^2^: 0.1230, p = 0.0182). In the ACS group, only dyslipidemia (β = 1.79, p = 0.0364) and HBP (β = − 2.37, p = 0.0183) shown an effect in the variation of IL-10 levels (adjusted R^2^: 0.046, p = 0.0226).

### Biochemical analysis

The biochemical parameters are described in Table 5. In the ACS group, most of the analytes were found within the reference ranges, probably due to the effect of the pharmacological treatment, and only glucose, HDL cholesterol, and CRP were found outside the reference values; while in the CG APO-A1 and APO-B were elevated, which could be explained by the heterogeneous presence of CRF in this group.

**Table 5:** Biochemical parameters in the studied groups.

Biochemical analyte	ACS, median (IQR)	CG, median (IQR)	Reference value	p
Triglycerides	89 (78–107)	118 (89.5–154)	< 250 ml/dL	0.0001
Cholesterol	117 (97–142)	180 (151.0–208.5)	150–199 mg/dL	< 0.0001
Glucose	**130** (99–150.2)	97 (88–122.2)	60–100 mg/dL	< 0.0001
LDL	41 (34–52)	89 (62–108)	< 130 mg/dL	< 0.0001
HDL	**16** (12–21)	46 (34–54)	> 40 mg/dL	< 0.0001
CRP	**24** (3–33.3)	2 (1–3)	Up to 5 mg/L	< 0.0001
APOA1	163.6 (149.3–179.3)	**198** (186.9–203.7)	94–178 mg/dL	< 0.0001
APOB	129 (110.4–157)	**159** (135.9–180.3)	63–133 mg/dL	0.0050

Results are shown in median (Interquartile range, IQR).

*ACS* acute coronary syndrome group, *CG* control group, *LDL* low density lipoprotein, *CRP* c-reactive protein, *HDL* high density lipoprotein, *APOA1* apolipoprotein-a1, *APOB* apolipoprotein-b. Significant values are in bold.

### Correlation analysis

In the CG, there was a positive association of IL-10 plasma levels with BMI (r = 0.3293, p = 0.0030) and an inverse correlation between *IL10* mRNA level and triglycerides (TGs) (r = − 0.6813, p = 0.0129). In the ACS group the level of *IL10* mRNA was inversely correlated with IL-10 plasma concentration (r =− 0.3191, p = 0.0331) and there was an inverse association between *IL10* mRNA and Creatine phosphokinase (CPK) (r = − 0.3757, p = 0.0283).

## Discussion

IL-10 is an immunomodulatory cytokine that have shown a protective role in formation, development, and stability of the atherosclerotic plaque that is the main trigger of ACS. The aim of this study was to establish whether − 1082 A > G, − 819 T > C and − 592 A > C variants located in the proximal promoter region of the *IL10* gene are associated with alterations on *IL10* gene expression and the plasma concentration in ACS patients. To the best of our knowledge, this is the first report describing *IL10* mRNA levels in PBMC and its correlation with IL-10 protein concentration in ACS patients.

In the present study, the median age in the ACS group was 64 years and the male sex was he most affected (72%). STEMI diagnosis represented 61% of ACS patients. Additionally, HBP was predominant over other CRF (71%). Our results were similar to those reported in RENASCA (National Registry of the acute coronary syndrome) study in Mexican population, in which men represented 75% and HBP was the most frequent CRF (60.5%). Also, STEMI presentation was the major clinical presentation of ACS^16^ in the RENASCA cohort.

In addition, all the quantified biochemical analytes showed significative differences between groups. In the ACS group most of the analytes were found within the established ranges, probably due to the effect of the pharmacological treatment. Particularly, we observed significative lower levels of HDL in the ACS group compared with the CG. Low HDL levels have been associated to the administration of β-blockers^17^ and in this study we documented a high proportion of ACS patients that consumed this drug (60%). Also, there is evidence of lowering plasma HDL particles due to the effect of high levels of IL-10 and this cytokine is a potent modulator of lipoprotein as well as triglyceride levels^18^. On this basis, we observed an inverse correlation of mRNA IL10 levels in regard to TGs levels (r = − 0.6813, p = 0.0129) in the CG. Additionally, hyperglycemia is frequently found in patients admitted because of acute myocardial infarction (AMI), regardless of a previously diabetes mellitus diagnosis and up to 20% of non-diabetic AMI patients had significant hyperglycemia^19^. Also, in the setting of ACS, Elevated CRP level may be related to the presence of ruptured atherosclerotic lesion and CRP concentration has shown to correlate with the extent of cardiac injury in the acute phase of AMI^20^. In the CG, these analytes were within normal range, except APO-A1 and APO-B that were elevated, which could be explained by the presence of cardiovascular risk factors in this heterogeneous group. This in concordance with a previous report that described the Mexican population with a high-risk profile, due to the agglomeration of several CRF^21^. These findings point out the necessity of stronger strategies for a better control of CRF in Mexican population in order to prevent cardiovascular complications such ACS which is the most important contributor of mortality in Mexico.

In addition to the effect of the traditional CRF, evidence of the role of the inflammatory response in cardiovascular disease (CVD) is well documented. IL-10, as the major immunomodulatory cytokine, has been extensively studied in CVD. The heritability of IL-10 secretion has been estimated to be 50% to 74% and for this reason, we included it in the study − 1082 A > G, − 819 T > C, − 592 A > C genetic variants located in the proximal promoter region of the *IL10* gene that have been associated with *IL10* gene transcriptional activity and IL-10 plasma levels. In this study, the analysis of the three gene variants showed that heterozygous genotypes were the most frequent in both groups, except for −  1082 A > G locus. There were no statistically significant differences in the allelic and genotypic frequencies and none of the genetic models of inheritance were associated with the risk of developing ACS. In contrast, it has been reported that the −  1082 AA genotype was associated with AMI risk in Italian individuals (OR 3.0 95% CI 1.78–5.04, p = 0.0001)^22^ and in South Indian population the − 1082 AA and − 592 CC genotypes were associated as risk factors for the development of ACS (OR 1.472, 95% CI 1.15–1.884, and OR 2.26, 95% CI 1.748–2.292, p = 0.0021; respectively)^13^. In East Asian individuals, − 1082 A allele was associated with lower predisposition to severe presentation of CAD (OR 1.24, 95% CI 1.02–1.50, p_dom_ = 0.03)^23^. Also, in Korean population, the C allele at loci − 592 and − 819 were associated with CAD risk (OR 1.37, 95% CI 1.03–1.82, p = 0.035 and OR = 1.35, 95% CI 1.02–1.80, p = 0.039)^24^. Moreover, in Mexican population, it has been previously expressed that the association of − 1082 A > G locus with silent myocardial ischemia (OR 1.77, 95% CI 1.06–2.98, pdom = 0.029) but no association with −  592 A > C neither −  819 T > C loci^25^. Finally, Fragoso et al. reported an association of − 592 C allele and CC genotype (OR 1.48, 95% CI 1.18–1.84, p = 0.0006, and, OR 1.56, 95% CI 1.11–2.19, p = 0.022, respectively) with ACS susceptibility in Mexican patients from the center of Mexico^26^. Regarding haplotypes, no significant differences between the distribution of haplotype frequencies were observed in the study groups. It was previously reported that in Mexican ACS patients from the center area of the country showed an increased frequency of ACC haplotype when compared to healthy controls (OR = 1.65, 95% IC 1.28–2.13, p = 0.0003) but a decreased frequency of ACA haplotype when compared to healthy controls (OR = 0.52, 95% IC 0.33–0.80, p = 0.006)^26^. In the present study we find no association of any variant neither haplotype with the susceptibility to ACS in Western Mexican individuals. It is well documented that Mexican population is formed by a great ethnogenetic diversity which could explain the observed differences among reports in Mexican population. It has been expressed that an increased Native American ancestry is seen in the central area of Mexico and the western area of Mexico has a more predominant European ancestry^27^.

Concerning IL-10 expression and secretion, in healthy individuals it has been reported that of those three variants, the position − 1082 A > G was an important genetic factor in the regulation of constitutive *IL10* mRNA and protein levels in healthy Spanish individuals^28^. Moreover, in CAD and AMI patients, the − 1082 A allele and AA genotype is associated with a lower expression of IL-10, and also with the susceptibility to the disease^22,29^. In the present study, we observed a lower concentration of IL-10 in patients carrying the − 1082 GG genotype in the ACS group; however, it was not significant (p = 0.0762) and *IL10* gene expression in PBMCs was not associated with genotypes of − 1082 A > G locus in any group. Nonetheless, since these three variants show a strong LD (Dʹ = 0.989, p < 0.0001), a more appropriate analysis could be haplotype approach. In the present study, when we analyzed the association of haplotypes with respect to IL-10 plasma levels, we did not find statistical significance in any group. Unfortunately, we were not able to carry out the haplotype analysis in association with *IL10* mRNA levels due to the low frequency of polymorphic alleles on mRNA sample size, but we do an analysis according to the dominant model of inheritance. In the same context, the − 819 T > C and − 592 A > C variants showed an association with *IL10* mRNA expression in the ACS group in the present study, there was a 0.4-fold lower gene expression of the cytokine when comparing to carriers of the CC genotype in both loci against levels of expression in heterozygous and polymorphic allele homozygous carriers only in the ACS group. Therefore, the − 819 T > C and − 592 A > C loci particularly influence *IL10* gene expression in the ACS individuals, without any significative difference of genetic variants with respect to IL-10 plasma levels. As suggested previously, the variants at − 819 T > C and − 592 A > C positions are more important in the regulation of basal mRNA levels in Western Mexican individuals than − 1082 A > G locus^12^ in accordance with the results of the present study. The findings of functional haplotype analyses have been diverse in the literature. It has been described that ATA haplotype is associated with lower cytokine expression^30^, whereas the CCA haplotype was associated with lower mRNA expression compared to ATA haplotype carriers^31^. But a layer of complexity is added when considering that *IL10* alleles are differentially transcribed in heterozygous individuals and that *IL10* haplotypes determine IL-10 production^32^. Though, a heritability of 50 to 74% in IL-10 secretion has been estimated, there is a marked interindividual variation in the production of the protein in healthy individuals. Such variation has been attributed to the relationship between genotype and transcriptional activity^28,31^. These variations could have implications in the assessment of physiological and/or pathological mechanisms associated with complex diseases such as ACS.

Also, we observed a negative correlation in *IL10* mRNA and IL-10 plasma levels (r = − 0.3191, p = 0.0331) in ACS group. The lack of concordance in the gene and protein expression of IL-10 has been previously reported both in healthy and diseased individuals. This could be attributed to post-transcriptional modifications, as well as protein secretion mechanisms that play a significant role as regulators of the expression of this cytokine^12,32^. Additionally, Bezafibrate, Amlodipine, Isosorbide and metformin influenced *IL10* mRNA levels, whereas bezafibrate, ASA and Clopidogrel had an effect over IL-10 protein levels (p = 0.0015 and p < 0.0001, respectively). Bezafibrates, PPAR agonists, have shown to inhibit the activation of inflammatory response genes such as tumor necrosis factor (TNF) and interferon (IFN)-β^33^ that are key signals for IL-10 production^34^. Also, since platelet activation, thrombosis and inflammation are closely related, antiplatelet drugs (ASA and Clopidogrel) have been shown to have direct or indirect anti-inflammatory effects both in vitro and in vivo, in addition to anticoagulant effects^35,36^. So far, there are few studies analyzing the association of these drugs with IL-10 levels and the exact immunomodulatory mechanisms have not been elucidated.

On the other hand, the association of IL-10 levels in the context of ACS pathology has been controversial. Although, the results regarding this association need to be considered carefully, since the individuals chosen for CG varies among reports and as well as the ACS type analyzed. The timing of blood sampling also could influence the observed results. In this study, when comparing the IL-10 levels between CG and ACS group, the latest showed higher levels. However, according to the RG, the levels of this cytokine in ACS group are similar to healthy individuals (RG) but in the CG the levels were under the reference range. In accordance with the protective role of IL-10^37^, this low concentration could imply that the Mexican population (particularly individuals over 45 years of age) could present a high risk of cardiovascular events and so, health strategies need to be implemented. Furthermore, we found that *IL10* gene expression in PBMCs differs depending on the clinical presentation of ACS, where the highest gene expression was found in patients with STEMI diagnosis, but plasma IL-10 concentration did not show significative differences among the clinical spectrum. Interestingly, it has been described that the proportion and type of immune cells is different depending on the presentation of ACS and even various cell subtypes have been associated with its severity^38^. This could imply that the *IL10* transcriptional activity depends on the ACS presentation, but the IL-10 half-life could not be affected by the clinical entity of ACS. For this reason, the *IL10* gene expression could be a more helpful biomarker than IL-10 plasmatic levels.

The focus on − 1082 A > G, − 819 T > C, and − 592 A > C SNVs in the *IL10* promoter region is due to their well-established roles in regulating *IL10* expression. Several relevant studies support the use of these three SNVs as markers of the proximal promoter region^14,22–25,31,32^. Additionally, numerous studies suggest that IL-10 may be associated with cardiovascular risk^13,24,25,29^. Functionally, *IL10* gene expression largely depends on these three SNVs and the haplotypes composed of them. Such haplotypes have been associated with high- or low-levels of *IL10* production^30–32^. However, the *IL10* locus (including the distal promoter, introns, exons, and 3′-UTR region) could potentially harbor additional functional SNVs that impact *IL10* expression. Also, the pattern of LD across this gene could be an excellent approach to discover other potential functional SNVs. Moreover, within the context of pathomolecular mechanisms, studying protein-DNA interactions could help to better understand the genetic regulation of the *IL10* gene. Exploring these areas could be an excellent option for further research.

### Limitations

A possible limiting factor in the present project is the pharmacological treatment prescribed to all patients. However, due to the acute nature of the disease, it is extremely difficult to include treatment-naive patients, which is why these factors were considered in the multivariate analysis. Additionally, it was not possible to perform comparative analysis of gene expression with respect to *IL10* promoter haplotypes due to the low frequency of polymorphic alleles on mRNA sample size analysis.

## Conclusion

The − 1082 A > G, − 819 T > C and − 592 A > C variants are not markers of susceptibility to ACS in the Western Mexican population. However, ACS patients showed a higher relative expression of *IL10* in PBMC than CG individuals, which could be mediated by the − 819 T > C and − 592 A > C loci since C/C homozygous carriers showed higher levels in the dominant inheritance model. Additionally, a decreased plasma concentration of IL-10 was observed in individuals from the CG, which could highlight the cardiovascular risk situation of the Mexican population. Furthermore, in patients with ACS, *IL10* mRNA levels were negatively correlated with plasma IL-10 levels. *IL10* mRNA levels were more informative markers of disease status than IL-10 plasma levels. mRNA expression was similar in CG and healthy individuals, but it was higher in ACS patients. On the opposite, ACS patients had similar IL-10 plasma values than healthy individuals, but CG values were lower than IL-10 levels in healthy individuals. Nevertheless, the results could be biased by pharmacotherapy. These findings could reflect the inflammatory process in individuals with ACS in addition to the genetic role in modulating the inflammatory response through the presence of gene variants in the promoter region of the *IL10* gene that impact the expression of the cytokine in pathophysiological conditions, which could provide the support for the investigation of the molecular mechanisms associated with the immunoinflammatory response that are specific to each ACS clinical presentation.

## Materials and methods

A case–control study was carried out. It included 300 individuals in the case group (ACS) and 300 individuals as control group (CG). The ACS group was formed by patients diagnosed with ACS according to guidelines of the American College of Cardiology^39^ from the Cardiology Service of the Western National Medical Center, Jalisco, Mexico. In the CG, unrelated volunteers with similar age to the cases (> 45 years) without personal history of ischemic cardiopathy were recruited from the general population. All participants were from Western Mexico in at least three generations (their own, parents and grandparents). There were excluded individuals diagnosed with other cardiac diseases as myocarditis, pericarditis, hypertrophic cardiomyopathy, valvular heart disease, Tako-Tsubo cardiomyopathy, cardiac trauma, congestive heart failure, among others), as well as overlap with non-cardiac diseases including pulmonary embolism, pulmonary infarction, pneumothorax, pleuritis, pneumonia, anemia, aortic dissection, aortic aneurysm, esophageal spasm, cerebrovascular disease. In both groups, individuals presenting genetic dyslipidemias and autoimmune diseases were not considered in this study. Written informed consent was obtained from all individual participants included in the study. Every individual enrolled in the present study was entirely informed about the risks and benefits implicated, and they were also notified about the possibility of publishing. All individuals agreed to participate, and they signed informed consent prior to the commencement of the study. Also, all mandatory laboratory health and safety methods have been complied. Additionally, this protocol conforms the ethical guidelines of the 1975 Declaration of Helsinki and guidelines developed by Council for International Organizations of Medical Sciences (CIOMS) in collaboration with the World Health Organization (WHO) solicited for the Ethics Committee of University of Guadalajara. This study protocol was accepted for the Ethics Committee and the Research Committee of University of Guadalajara under the number of registry CI-01614.

In the ACS group, the clinical record was consulted for the obtention of clinical data as troponins levels, creatine kinase (CK), and creatine kinase MB fraction (CK-MB). All clinical parameters such as height, weight, comorbidities, lifestyle, and family history of myocardial infarction were obtained by questionnaire in both groups. Biochemical profile measurement was performed on plasma from patients and control subjects using Biosystems reagents (Biosystems S.A). All blood samples were obtained after all night fasting. The biochemical analytes measured in both studied groups were glucose, triglycerides, total cholesterol, high-density cholesterol (HDL), low-density cholesterol (LDL), C-reactive protein (CRP). Drugs that were part of the pharmacological treatment in the ACS group were: Amlodipine, Acetyl Salicylic Acid (ASA), clopidogrel, Enoxaparin, Amiodarone, beta-blockers, Angiotensin converting Enzyme Inhibitors (ACEI), Losartan, Furosemide, Spironolactone, statins, bezafibrates, isosorbide, metformin, and insulin. In the CG the medications that the individuals consumed were ASA, losartan, glibenclamide, metformin, statin and bezafibrate.

The extraction of genomic DNA (gDNA) was performed from total peripheral blood leukocytes, using the modified Miller’s method^40^. The identification of *IL10* gene promoter variants − 1082 A > G, − 819 T > C and − 592 C > A was carried out by allelic discrimination using TaqMan^®^ probes (catalog No. C_1747360_10, C_1747362_10 and C_1747363_10, respectively; Applied Biosystems, USA) with VIC^®^ and FAM™ fluorochromes and two specific primers for each of the variants. VIC® is the probe to detect allele 1 (wild type) and FAM ™ is the probe to detect the sequence of allele 2 (polymorphic). Genotyping was carried out in a Light Cycler^®^ 96 instrument (Roche, USA). As quality control, 25% of the samples were repeated and full matching was obtained.

It is worth noting that the variants are also referred as: rs1800896 T > C (Reference SNP ID notation assigned by dbSNP), −  1082 A > G (Legacy nomenclature), or *IL10* NG_012088.1:g.3943A > G (nomenclature urged by the Human Genome Variation Society, HGVS); rs1800871 A > G, − 819 T > C, or *IL10* NG_012088.1:g.4206 T > C; and rs1800872 T > G, − 592 C > A, or *IL10* NG_012088.1:g.4433A > C. According to our population sample, the reference (C) allele was the most common in the case of -819 T > C and − 592 C > A variants. In this report we considered the C allele of both variants as the wild type due to its higher frequency in our study population.

Peripheral blood mononuclear cells (PBMC) of 28 SCA patients and 28 CG individuals, matched by age, were isolated using Lymphoprep reagent according to the manufacturer instructions (catalog no. 1858, Serumwerk, GE). Total RNA was extracted from PBMC according to Chomcyznski and Sacchi method^41^. mRNA purity and concentration were determined by spectrophotometry (NanoDrop Lite Spectrophotometer, Thermo Scientific, USA) using the A260/A280 ratio. To perform the retrotranscription step*,* 1 ng of total RNA was reverse transcribed using oligo-dT and M-MLV reverse transcriptase according to the manufacturer (Promega, USA). For gene expression analysis, we chose the samples that represent the clinical spectrum and to pair by age the CG and ACS group.

The determination of *IL10* gene expression was performed by quantitative PCR (qPCR) using TaqMan probes (Applied Biosystems, USA). The primers used for *IL10* gene expression assay were forward: 5ʹ -GAGAACAGCTGCACCCACTTC-3ʹ and reverse: 5ʹ-GGGCATCACCTCCTCCAGGTAA-3ʹ (hs00961622_m1, catalog no. 4331182, applied biosystems, USA). *GAPDH* gene was used as reference and the primers for this gene were: forward: 5ʹ -GAGTCAACGGATTTGGTCGT-3ʹ and reverse: 5ʹ -TTGATTTTGGAGGGATCTCG-3ʹ (hs02786624_g1, catalog no. 4331182, applied biosystems, USA). qPCR was performed in a Light Cycler^®^ 96 instrument (Roche, USA), under the conditions indicated in the Taqman Gene Expression Assay protocol. All samples were run in triplicate. For gene expression assays, we followed the recommendations stated in the Minimum Information for Publication of Quantitative Real-Time PCR Experiments (MIQE) guidelines^42^. To determine the *IL10* mRNA relative expression we used the ${2}^{-\Delta \Delta Cq}$ method after data validation as suggested by Livak and Shmittgen^43^.

Quantification of IL-10 protein was determined in 157 ACS and 126 GC plasma samples using a high sensitivity Enzyme-Linked Immunoenzyme Assay according to the manufacturer’s instructions (human IL-10 High Sensitivity ELISA Kit, catalog no. BMS215HS, Invitrogen,USA). The range of detection of the assay was 0.16–12.70 pg/mL, and the sensitivity of the assay was 0.05 pg/mL. As quality control, 10% of the samples were double measured and a coefficient of variation < 10% was obtained. As our CG individuals presented diverse cardiovascular risk factors that represent the source population that give rise cases^44^ our controls were not healthy. Thus, we calculated the baseline levels of IL-10 plasma levels in a pool of 78 healthy western Mexican individuals that conformed a reference group (RG). The quantification of IL-10 plasma levels in the RG was made for two reasons: first, the levels of diverse analytes can vary among populations cases^44^ and it has been recommended to obtain specific values for each population. Second, as our CG was formed by non-healthy individuals as they presented a variety of cardiovascular risk factors to adequately represent the source of cases according to the epidemiological design of the study cases^44^, we aimed to establish the general population baseline IL-10 levels. This sample population included 39 males and 39 females with a median age of 28 (24–36 years). The median (IQR) concentration of IL-10 in the RG was 1.5577 (1.0441–1.8704) pg/mL. We used the IQR as a reference range of IL-10 baseline levels. For the CG and the ACS group we chose the samples that represent the clinical spectrum by age and gender, and samples that we had the gene expression data.

### Statistical analysis

The Kolmogorov–Smirnov test was performed to test the normality of the variables and Levene test was used to assess variance in all variables. In all analysis, a value of p < 0.05 was taken as statistically significant in all analysis. Due to the non-gaussian distribution of data, non-parametric tests (*U*-Mann–Whitney, Kruskal–Wallis, Spearman correlation) were performed. Qualitative data were analyzed using X^2^. The allelic and genotypic frequencies of the variants were obtained by direct count. Hardy–Weinberg equilibrium (HWE) analysis was carried out in each group, and it was evaluated for each locus. HWE as well as allelic and genotypic association analysis were analyzed using X^2^ and the association was expressed as odds ratio (OR) with risk estimates in the range 95% confidence interval (CI) and its p-value. Haplotype inference was performed in SHEsis web-based platform^45^. Linkage Disequilibrium (LD) was described as Dʹ corrected coefficient as well as its corresponding p-value. The comparison of the observed and expected values of each haplotype was performed using the X^2^ test. Only haplotype frequencies greater than 3% were considered to reduce bias due to the effect of sample size.

Binary logistic regression (qualitative dependent variable) or linear regression (quantitative dependent variable) analysis was performed to identify the relationship between each of the covariates (clinical variables) and the dependent variable.

## Data Availability

The datasets analyzed during the current study including accession information for the raw genotyping data are available from the corresponding author on reasonable request.
